# Insights into malaria pathogenesis gained from host metabolomics

**DOI:** 10.1371/journal.ppat.1008930

**Published:** 2020-11-12

**Authors:** Heather N. Colvin, Regina Joice Cordy

**Affiliations:** 1 Department of Biology, Wake Forest University, Winston-Salem, North Carolina, United States of America; 2 Department of Microbiology and Immunology, Wake Forest School of Medicine, Winston-Salem, North Carolina, United States of America; University at Buffalo School of Medicine and Biomedical Sciences, UNITED STATES

## Introduction

Malaria is a devastating disease caused by the protozoan parasite *Plasmodium*. The most common *Plasmodium* species that infect humans are *Plasmodium falciparum* and *Plasmodium vivax*, which together cause the vast amount of the disease’s morbidity and mortality worldwide [1]. From a clinical perspective, *Plasmodium* causes a spectrum of disease ranging from asymptomatic to severe. From a biochemical perspective, malaria involves an interconnection of host and parasite through a shared resource environment, resulting in the exchange of nutrients and signaling molecules. Within the bloodstream of *Plasmodium*-infected hosts, perturbations in the levels of various metabolites occur, including amino acids, lipids, fatty acids, sugars, and heme metabolites [2].

Metabolomics is a robust tool to study host–pathogen interactions. In-depth analysis of metabolism and the associated by-products and pathways can be viewed in snapshots of time, and the biochemical fingerprints contribute greatly to our understanding of the complex interaction between hosts and pathogen [3]. Metabolomics utilizes methods such as nuclear magnetic resonance (NMR) spectroscopy, liquid chromatography–mass spectrometry (LC–MS), or gas chromatography–mass spectrometry (GC–MS) to identify small weight molecules known as metabolites. Analyses can be performed on biological fluids and tissues (e.g., plasma and urine), volatile organic compounds (VOCs, e.g., odor from breath or skin), and cell cultures as either untargeted or targeted, the former outputting a vast dataset based on chemical features (e.g., mass-to-charge ratio) and the latter including chemical annotations based on reference compounds. Metabolite results can be further analyzed for biochemical involvement using publicly available databases such as Malaria Parasite Metabolic Pathways (MPMP) [4] and Kyoto Encyclopedia of Genes and Genomes (KEGG) [5], to name a few.

Within the past several years, numerous published works have emerged that employ metabolomics methodology toward the goal of better understanding malaria infection. These metabolome studies have both confirmed previous biological findings that were determined through careful molecular and cellular experimental work as well as shed light on new findings for which the biological underpinnings are still unclear. Prior reviews have provided an overview of the metabolism of *Plasmodium* from a host–parasite interaction viewpoint [6,7] as well as covering how host metabolites may contribute to malaria transmission [8]. Here, this review aims to summarize the status of our knowledge about metabolic fluctuations that occur in the host during malaria infection that may relate to malaria pathogenesis, immunity, and diagnosis. In particular, we focus on amino acid, lipid and fatty acid, and red blood cell (RBC)-related alterations in the bloodstream of hosts during malaria infection and how this compares to other diseases. We also discuss metabolites produced by the parasite and by the gut microbiota, respectively, and discuss the potential for metabolite-based biomarkers to aid in malaria diagnostics.

### Bloodstream amino acid and glucose perturbations in malaria

**Fig 1:**
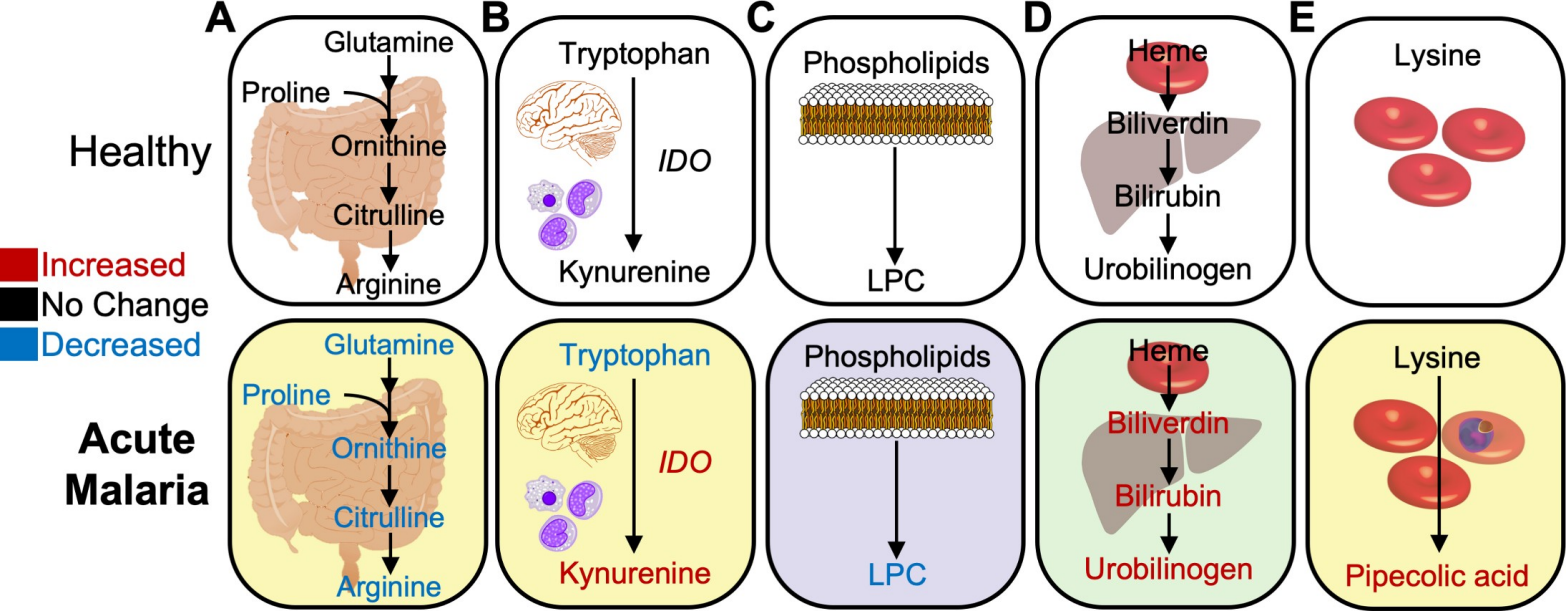
Examples of metabolic pathways perturbed in the host during acute malaria. Biochemical pathways of amino acids (yellow), lipids (purple), and heme (green) found to be either increased (red) or decreased (blue) in acute malaria as compared to healthy individuals. (A) Amino acids involved in the de novo biosynthesis of arginine are globally decreased during malaria [9,18,21]. Metabolic changes associated with malaria also include (B) elevated conversion of tryptophan to kynurenine via *IDO* enzyme [9,11,20,28], (C) depleted LPC from phospholipids [9,11,32], and (D) increased heme products indicating hemolysis and hemoglobin degradation [9–11]. (E) Lysine catabolism into pipecolic acid is also detected in *Plasmodium* infections [33–35]. IDO, indoleamine 2,3-dioxygenase; LPC, lysophosphatidylcholine.

A significant depletion of amino acids occurs in the bloodstream of *Plasmodium*-infected hosts, and a number of studies have characterized these perturbations [9–22]. Of these, arginine, glutamine, and tryptophan have received the most attention in recent studies due to the direct clinical consequences when either are decreased. Low levels of arginine in the bloodstream during malaria may underlie downstream consequences of impaired vasodilation, endothelial disruption, and reduced nitric oxide production [16]. While depletion of host arginine could derive in part from parasite-specific processes (e.g., elevated *Plasmodium* arginase activity [19]), experiments using murine and nonhuman primate models paired with analyses of human samples have demonstrated simultaneously diminished levels of arginine and its biosynthetic pathway metabolites (e.g., ornithine and citrulline) in the blood of malaria-infected hosts [9,18,21] (Fig 1A). This work suggests that arginine depletion results, at least in part, from a block in host production, in addition to parasite arginase activity. So, why is arginine in low supply? A probable cause is the limited bioavailability of precursors for arginine biosynthesis, including glutamine and proline, which decrease in parallel during malaria [21].

Glucose and glutamine are important precursors for energy production by both host and parasite. *Plasmodium* relies primarily on glycolysis for ATP production, and parasites take up glucose in large amounts during their development, leading to increased lactate production [23]. *P*. *falciparum*–infected hosts may have increased lactate in the bloodstream, a condition called metabolic acidosis [24], and glycolysis pathway metabolites have been shown to be particularly perturbed in the bloodstream of *P*. *falciparum* malaria patients [25].

For ATP production, both *Plasmodium*-infected RBCs and host immune cells also consume glutamine, which is fluxed into the tricarboxylic acid (TCA) cycle [23,26,27], and plasma glutamine levels are depleted in the human host in both falciparum and vivax malaria [9,21,22,28]. In addition to impacting arginine biosynthesis (Fig 1A), low plasma glutamine has been associated with severe malarial anemia in children with *P*. *falciparum* [29] and with impaired humoral immunity in a murine model of severe malaria [27]. Conversely though, inhibiting glutamine metabolism is associated with increased survival in a murine model of late stage cerebral malaria (CM) via reducing immune-mediated pathology in the brain [30]. Glutamine may therefore be a “double-edged sword” in the pathogenesis of malaria due to its opposing effects on these different manifestations of disease.

Also implicated in severe malaria is the enzymatic conversion of tryptophan to kynurenine, which is elevated during severe malaria, resulting in the production of neurotoxic metabolites (e.g., quinolinic and kynurenic acid), which are thought to play a role in CM [11,20] (Fig 1B). A decrease in indolepropionate, a neuroprotective derivative of tryptophan, is also observed in the bloodstream of humans with CM and may further contribute to neurological dysregulation [11]. Elevated production of kynurenine from tryptophan is not, however, specific to neurological diseases like CM, and similar perturbations have been observed in non-CM malaria [9,20,28]. Elevated kynurenine also indicates elevated indoleamine 2,3-dioxygenase (IDO) enzymatic activity and the initiation of the host’s immunotolerant responses. While tryptophan catabolites may have a neurotoxic role, the catabolism of tryptophan is likely driven by the host’s acute response to malaria, which includes both pro-inflammatory and anti-inflammatory tolerogenic programs [31].

### Bloodstream lipid and fatty acid perturbations in malaria

Acute falciparum and vivax malaria infections in humans coincide with a reduction in monounsaturated fatty acid–containing phospholipids, a reduction of lysophosphotidylcholines (LPCs), and an elevation in fatty acyl carnitines [9,10,32]. This pattern suggests an increase in beta oxidation of fatty acids in mitochondria as a means of energy production. Phospholipase A_2_ (PLA_2_) is a host hydrolytic enzyme that acts on phospholipids to release lysophospholipids and free fatty acids. In humans with falciparum malaria, PLA_2_ activity has been associated with an enrichment of a particular downstream product, arachidonic acid (AA), which modulates inflammation [11]. Prior metabolomic studies correlated brain volume with downstream PLA_2_ products, suggesting that this pathway may play a key role in the pathogenesis of CM [12].

LPCs are reduced in humans with acute malaria (Fig 1C) [9,11,32], which may result in part from host metabolic processes that are altered during acute infection states. The parasite may also play a role in depleting host plasma LPC, as parasites take up lipids and fatty acids from their environment to build their own membranes. Regardless, low levels of certain glycerophospholipids, such as LPC, in plasma may promote conversion to *Plasmodium* gametocyte stages, which are required for malaria transmission [33,34]. This metabolic perturbation may therefore play a critical role in perpetuating the life cycle of the parasite.

### Red blood cell–related alterations in malaria metabolome

Malaria is associated with a vast loss of RBCs due, in part, to parasite-mediated lysis, with hemoglobin and free heme being released in the process. Heme containing iron induces oxidative stress on RBCs [35] and likely contributes to further lysis of the host’s uninfected RBCs during malaria infection (e.g., “bystander effect”) [36]. Metabolic processes are subsequently mounted by the host in an attempt to detoxify heme. Free heme converts to bilirubin in the liver, spleen, and bone marrow using biliverdin as an intermediate, or in the intestine, using urobilinogen as an intermediate. Multiple reports document elevated biliverdin, bilirubin, L-urobilin, and I-urobilinogen in the plasma of human malaria and its animal models [9–11], presumably resulting from the host’s response to free heme during malaria infection (Fig 1D).

### Metabolic changes during mild or asymptomatic malaria

Although mild or asymptomatic malaria is generally tolerable compared to its severe form, low-level chronic parasite carriage is still associated with changes in host metabolism. Studies of low parasitemia in human falciparum malaria and its nonhuman primate model include altered energy metabolism pathways and reductions in multiple lipids, including sphingomyelins [9]. Sphingolipid metabolism has been thought to play a role in signaling related to immune responses and vascular integrity and possibly aid in controlling the infection [37]. Aside from bloodstream changes, enriched levels of certain VOCs have also been detected in the skin odor of humans with asymptomatic *P*. *falciparum* malaria, including ethylbenzene, which has been shown to be a mosquito attractant [8,38].

### Comparable metabolic dysregulation in other blood diseases

Some alterations in host bloodstream metabolite levels during malaria are also common among hosts with other diseases. For instance, decreased arginine levels has received much attention in malaria, but this amino acid is also markedly decreased in hemolytic anemia and sepsis, pointing to similar host responses among these conditions [39]. Additionally, altered levels of kynurenine [40], PLA_2_ [41], and LPC [42] have also been reported in sepsis, highlighting the possibility of common host-mediated metabolic responses across acute bloodstream infections.

### *Plasmodium-*derived metabolites identified using metabolomics

During its intraerythrocytic development, *Plasmodium* derives most amino acids from hemoglobin degradation. In the process, *Plasmodium* produces various by-products including pipecolic acid, a catabolite of lysine (Fig 1E). Pipecolic acid is detected in *in vitro P*. *falciparum* cultures, murine malaria models, and humans with *P*. *falciparum*, but not in uninfected RBC cultures or in humans without malaria infection [43–45]. Other metabolites potentially generated by the parasite include VOCs pinene and limonene, which may derive from *Plasmodium*’s isoprenoid biosynthetic pathways and are detected in *P*. *falciparum* cultures and breath of humans with falciparum malaria [46]. Metabolites in the alpha-linolenic acid pathway, commonly found in plants, have also been found in both *P*. *falciparum* cultures and plasma from infected patients [47].

Additional metabolite signatures have been identified through untargeted metabolomics approaches of *P*. *falciparum* in vitro culture, including 3-methylindole, succinylacetone, S-methyl-L-thiocitrulline, and O-arachidonoyl glycidol [48]. While about half of the detectable metabolic features measured through this untargeted approach could be mapped to KEGG metabolic pathways for human and *Plasmodium*, over 500 metabolic features detected in *Plasmodium* culture could not be matched to these databases, and many of these metabolite identities are yet to be determined. These may represent interesting candidates for future research.

### Host gut microbe impact on the metabolome during malaria infection

Plasma metabolomics applied to humans experiencing metabolic acidosis during falciparum malaria has revealed the presence of organic acids potentially of bacterial origin, including diaminopimelic acid, a component of gram-negative bacterial cell wall. Elevated diaminopimelic acid was observed concomitant with a depletion in L-citrulline [17], which plays a role in maintaining intestinal barrier function [49]. Gut barrier integrity can be lost during malaria, enabling gut microbes to translocate into the bloodstream. As levels of bacteria-associated metabolites in plasma were associated with elevated disease severity [17], this suggests a potential link between the presence of these metabolites in the bloodstream and pathological processes involving gut bacteria.

### Metabolic biomarkers provide potential for novel malaria diagnostic tests

Implementation of point-of-care diagnostics guided by metabolome findings could aid in the diagnosis and appropriate treatment of malaria and help to differentiate it from non-malarial febrile illnesses. A current rapid diagnostic test (RDT) for malaria includes detection of histidine-rich protein 2 and 3 (*hrp2/3*), which unfortunately has failed to detect *hrp2/3* gene deletion strains of *Plasmodium*, which may be rising in prevalence [50]. Furthermore, current tests do not indicate disease severity which, if incorporated, could provide prognostic benefit to current RDTs and allow for prompt treatment and better resource allocation for those likely to develop severe disease [51]. Studies comparing the metabolome of malaria with non-malarial febrile illnesses have identified both common and distinct features of malaria [9,52]. RDTs that include both *Plasmodium* infection markers (e.g., elevated pipecolic acid [45] and pinene [46]) and disease severity markers (e.g., depleted arginine [16], glutamine [29], and citrulline [17]) could have diagnostic and prognostic benefit.

## Conclusion

Dynamic perturbations in host metabolites occur in individuals infected with *Plasmodium*. Some of these metabolic signatures overlap with other acute infectious and inflammatory responses, such as sepsis, which is characterized by catabolic distress involving a breakdown of carbohydrates, lipids, and protein stores. Overall, a notable dysregulation of amino acids and lipids occurs during *Plasmodium* infection, likely resulting from catabolic and anabolic processes for immune cells and parasites alike. Although there have been significant metabolic findings to elucidate host and pathogen interactions during malaria as highlighted in this review, there are still many areas to investigate to further understand metabolic roles during infection.
